# Membranes for Redox Flow Battery Applications

**DOI:** 10.3390/membranes2020275

**Published:** 2012-06-19

**Authors:** Helen Prifti, Aishwarya Parasuraman, Suminto Winardi, Tuti Mariana Lim, Maria Skyllas-Kazacos

**Affiliations:** 1 School of Chemical Engineering, The University of New South Wales, UNSW Sydney, NSW 2052, Australia; Email: m.kazacos@unsw.edu.au; 2 School of Civil and Environmental Engineering, Nanyang Technological University, Singapore 639798, Singapore; Email: Aishwarya@ntu.edu.sg (A.P.); suminto@ntu.edu.sg (S.W.); 3 School of Life Sciences and Chemical Technology, Ngee Ann Polytechnic, Singapore 599489, Singapore

**Keywords:** energy, redox flow batteries, membrane, stability, ionic conductivity

## Abstract

The need for large scale energy storage has become a priority to integrate renewable energy sources into the electricity grid. Redox flow batteries are considered the best option to store electricity from medium to large scale applications. However, the current high cost of redox flow batteries impedes the wide spread adoption of this technology. The membrane is a critical component of redox flow batteries as it determines the performance as well as the economic viability of the batteries. The membrane acts as a separator to prevent cross-mixing of the positive and negative electrolytes, while still allowing the transport of ions to complete the circuit during the passage of current. An ideal membrane should have high ionic conductivity, low water intake and excellent chemical and thermal stability as well as good ionic exchange capacity. Developing a low cost, chemically stable membrane for redox flow cell batteries has been a major focus for many groups around the world in recent years. This paper reviews the research work on membranes for redox flow batteries, in particular for the all-vanadium redox flow battery which has received the most attention.

## 1. Introduction

Concern over environmental degradation and climate change associated with fossil fuel based electricity generation has prompted most countries to restructure their electricity generation and distribution in particular, towards increasing electricity generation from renewable energy sources. 

Renewable energy is intermittent in nature and thus the electricity generated from this source is not dispatchable, leading to unpredictable matching between supply and demand. Therefore energy storage is needed to prevent blackouts caused by unbalanced supply and demand. Besides, energy storage can also assist the utility planner to bridge the gap between the drops in the rate of electricity generated by renewable energy sources due to cloud cover over photovoltaic panel or reduced wind speed and the ramp up rate of gas powered peaking plants which normally take about 15 minutes to ramp up their power output. There are many types of energy storage technologies including pumped hydro, compressed air, fly-wheels and electrochemical systems such as fuel cells and redox flow batteries. Among these energy storage technologies, redox flow battery is considered the best option for medium to large scale storage owing to an excellent combination of energy efficiency, capital cost and life cycle costs without specific site requirement [1].

**Figure 1:**
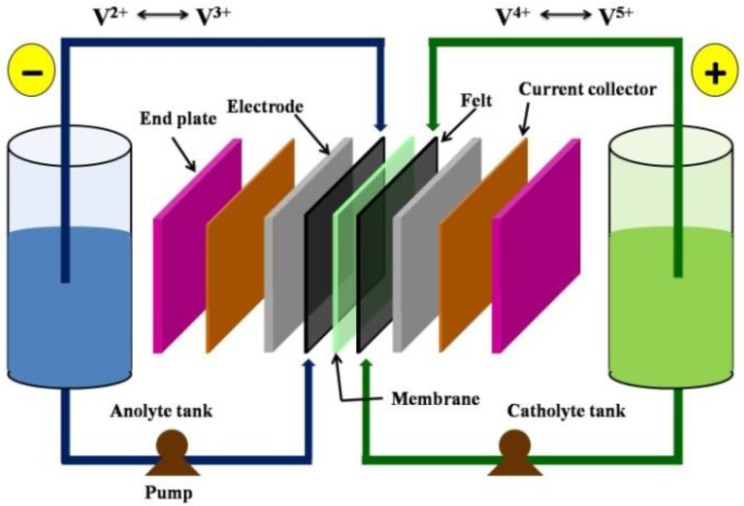
Schematic of a Vanadium redox flow battery (Adapted from [2]).

Redox flow battery uses two soluble redox couples as electroactive materials to store energy via oxidation and reduction reactions. In a typical set-up, the redox flow battery consists of 2 electrolyte reservoirs from which the electrolytes are circulated by pumps through an electrochemical cell stack comprising of a number of cells connected in series or parallel to enable reaction taking place at inert electrodes. Typically, each cell comprises of anode, cathode and an ion exchange membrane separator to allow diffusion of ions across the membrane while preventing the cross-mixing of the electrolyte solutions from these 2 reservoirs. Figure 1 shows the diagram of a vanadium redox flow battery (VRB).

During charging, by applying an external power source at the terminals, the discharged form of the redox species in each half cell electrolyte is converted to the charged form. During discharge, electron flows between the redox species thus converting the chemical energy into electricity. Consequently, the concentration of the active redox species and electrolyte volume will determine the energy capacity of the system while the number of cells in the stack and electrode area will determine the system power. The redox flow battery therefore acts more like a regenerative fuel cell than a conventional battery [3]. 

Of all the redox flow batteries developed to date, only the all vanadium redox flow battery developed at the University of New South Wales [3,4] has received the most attention due to its high energy efficiency of over 80% in large installations and a long cycle life. The major issues encountered by other redox flow batteries such as iron/chromium, iron/titanium, polysulfide bromine (also known as Regenesys) redox flow batteries include cross contamination of electrolytes due to different redox couple species used in each half-cell and the lack of development of an ideal membrane. The all vanadium flow battery uses the same vanadium element in both half cells which avoids the problems of cross contamination of the two half-cell electrolytes during long term usage. This helps in reducing the capital and maintenance costs, minimizes waste disposal and provides a greater ease of operation. Other attractive features include the ability to use underground electrolyte storage tanks leading to minimal footprint and temperature fluctuations in extreme climates. Furthermore, the redox flow battery can be recharged either conventionally or with mechanical refueling at a suitable refueling station, making it attractive for electric vehicles applications [2]. 

The original all-vanadium redox flow battery, also known as Generation 1 (G1) VRB uses a solution of vanadium in sulphuric acid in both half cells with V^2+^/V^3+^ redox couple operating in the negative half-cell and the VO^2+^/VO_2_^+^ redox couple in the positive half-cell. During charging, V^3+^ is reduced to V^2+^ at the negative electrode while VO^2+^ is oxidized to VO_2_^+^ at the positive electrode. These reactions are reversed during cell discharge. The maximum vanadium ion concentration that can be employed for wide temperature range operation is typically 2 M or less [5,6,7,8,9]. This concentration is equivalent to an energy density of around 25 Wh/kg and represents the solubility limit of the V(II) and/or V(III) ions in sulphuric acid supporting electrolyte at temperatures below 5 °C and the stability of the V(V) ions at temperatures above 40 °C [10]. Furthermore, significantly lower electrolyte concentrations are needed in many geographic locations where the climate is more extreme and temperatures go below zero degrees in winter. In such climates, vanadium sulphate concentrations as low as 1 M may be needed to avoid precipitation and this leads to further reduction in overall energy capacity.

Recent studies showed the use of a halide supporting electrolyte that allows the preparation of vanadium electrolyte with a concentration of up to 4 M leading to the development of Generation 2 (G2) VRB or the vanadium bromide redox flow battery (V/Br) [4]. The G2 VRB employs a vanadium bromide/chloride mixed electrolyte in both half-cells. Since the bromide/polyhalide couple has a less positive potential than the V (IV)/V (V) couple, the bromide ions will preferentially oxidize at the positive electrode during charging. The positive half-cell thus utilizes the Br^−^/ClBr_2_ or Cl^−^/BrCl_2_^−^ redox couple while the negative half-cell utilizes the same V^2+/^V^3+^ redox couple reaction, similar to the G1 VRB. Since the same electrolyte are used in both half cells, the G2 V/Br shares all the benefits of the G1 VRB technology, particularly with the fact that cross contamination is eliminated, resulting in solutions having an indefinite life [3]. The additional benefit of G2 VRB is the ability to use electrolytes up to a concentration of 4 M and hence having the potential to double the energy density of the G1 VRB and thus extending its energy storage usage for mobile applications. The G2 VRB can also operate at higher temperature ranges (0–50 °C), thus eliminating the thermal precipitation reaction for V (V) and increasing the solubility limits for the other vanadium ions. More recently, researchers at the Pacific Northwest Laboratories demonstrated a significant increase in energy density and a stable temperature range by utilizing a mixed H_2_SO_4_/ HCl supporting electrolyte that can optimize the solubilities of each of the vanadium oxidation states, allowing up to 2.7 M Vanadium solutions to remain stable over the temperature range of 0–50 °C [11]. Both electrolyte improvements will enable installations in extreme climates such as Northern China, Canada and Scandinavian countries that may not be suitable for the G1 VRB electrolyte. 

In both G1 and G2 VRB system, the key material is the membrane as it defines the performance and economic viability of the batteries. In fact, membrane is identified as the stumbling block towards commercialization of many redox flow batteries as it can contribute up to 20% to the overall battery system cost. Finding and developing a suitable membrane to provide the right balance between performance and cost has now become a main focus for redox flow cell developers around the world. Extensive research on developing suitable membrane material has been carried out since 2005 and earlier reviews by Li *et al.* [12] and Schwenzer *et al.* [12,13] focus on G1 VRB membranes which may not be applicable to the G2 VRB systems. The present paper focuses on all aspects of ion exchange membrane including their classification, structure, methods of preparation and their application in redox flow batteries, particularly for the G1 and G2 VRB. Future direction with respect to the development of next generation membrane materials is also included in this review.

## 2. Ion Exchange Membranes

The membrane is an important component in a redox flow cell. A great amount of effort is generally put into selecting a suitable membrane for redox flow systems. In vanadium redox systems, an ideal membrane should offer the following characteristics: good chemical stability under acidic conditions, resistance to the highly oxidising environment of the positive half cell electrolyte, low electrical resistance, low permeability to the vanadium or polyhalide ions, high permeability to the charge carrying hydrogen ions, good mechanical properties and low cost [14]. In addition to the above mentioned characteristics, an important property that an ideal membrane must possess, is the ability to prevent the preferential transfer of water from one half cell to the other, as this results in flooding of one half cell while diluting the other [15]. In redox flow cells, the function of the membrane is to prevent cross mixing of the positive and negative electrolytes and the short circuiting of the two half cell electrodes while allowing the transfer of ions to complete the circuit during the passage of current [15,16,17]. The membrane has been identified as one of the main obstacles in the commercialisation of many redox flow cells [18]. Ion exchange membrane dependent processes were first developed by Ostwald in 1890, who studied semi permeable membranes and discovered that a membrane can be impermeable to any electrolyte if it is impermeable to either its cation or anion [19]. Ion exchange membranes are sheets, ribbons or tubes, which separate two fluids and are capable of ion exchange [20]. Ion exchange membranes are very similar to ion exchange resins, the difference arising from the mechanical requirements of the membranes. Ion exchange resins are dimensionally unstable, as cation exchange resins are often brittle, whilst anion exchange resins are soft [21]. These mechanical properties arise from the lack of backing material that gives membranes the necessary strength and dimensional stability [21]. The membranes are made up of cross-linked linear polymer chains, which form a three-dimensional network. Without the cross-linking, the membrane would dissolve in water forming a polyelectrolyte solution. Ion exchange membranes, like the resins, have fixed ion functional groups and oppositely charged counter ions, present in sufficient numbers to render the whole exchanger electrically neutral. 

**Figure 2:**
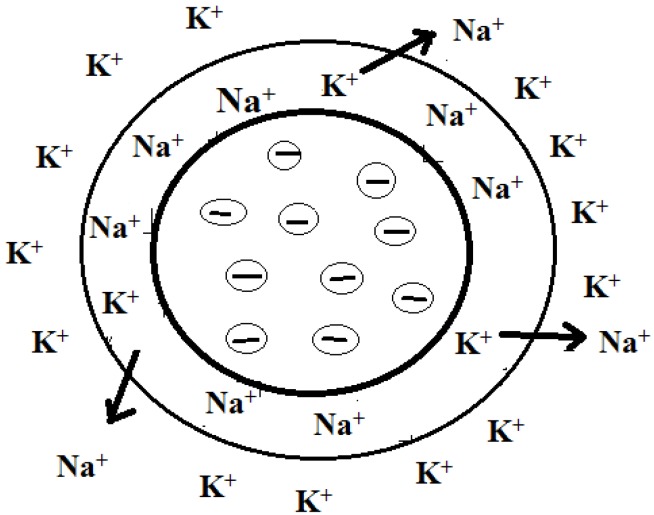
Schematic representation of an ion exchange process.

The ionic functional groups are the exchange sites which are capable of forming an electrostatic bond with an ion of opposite charge [22]. The process of ion exchange occurs when the mobile counter ions are replaced by other ions with the same charge from solution. Ion exchange is a reversible and stoichiometry process, with the displacement of one ionic species by another on the exchanger [23]. The ease with which the ion may be replaced depends on the strength of the bond, which varies similar to the dissociation of strong and weak electrolytes [22]. Figure 2 depicts an ion exchange process.

Research into the basic principles of ion exchange continued with the work of Donnan [24], Sollner and others. However, it was not until 1950 that commercial applications began to expand with the stable, highly selective and low electric resistance membrane of Juda and McRae (Ionics Inc., Cambridge, MA, USA) [25]. Today, ion exchange membranes are applied in desalination, in the treatment of industrial effluents, in the food, manufacturing and pharmaceutical industries. Their applications range from removal of heavy metals to the removal of salt in an advanced continuously operated upflow microbial desalination cell [26]. In recent years, research in membrane technology has greatly focussed on battery applications. In vanadium redox batteries, the most widely investigated membranes have been perfluorosulfonic acid polymers such as DuPont’s Nafion^®^ that have good stability to the highly oxidising V (V) solution in the charged positive half cell electrolyte [13]. The high cost and relatively poor selectivity of Nafion membranes for the vanadium ions, have however, precluded their use in commercial systems. 

## 3. Types of Ion Exchange Membranes

Traditionally, ion exchange membranes are classified into cation exchange membranes and anion exchange membranes depending on the type of ionic functional groups attached to the membrane matrix [19]. Cation exchange membranes contain negatively charged groups such as –SO_3_^−^, –COO^−^, –PO_3_^2−^, –PO_3_H^−^, –C_6_H_4_O^−^. They allow the passage of cations, such as the Na^+^ shown in Figure 2, but are non-permeable to anions such as Cl^−^. Anion exchange membranes have positive functional groups such as –NH_3_^+^, –NRH_2_^+^, –NR_2_H^+^, –NR_3_^+^, –SR_2_^+^, and thus allow the passage of anions. Composite membranes, called bipolar membranes, can be produced to contain a cation selective layer (with negative fixed ionic groups) and an anionic layer (with positive ionic groups). Apart from polymeric ion exchange membranes, ion exchange membranes are also prepared from inorganic material like zeolites, betonite or phosphate salts. However, they have certain disadvantages because of their high cost and relatively bad electrochemical properties [19]. 

In general, membranes can be classified based on a lot of factors like nature of the material, morphology, pore size, the driving force and their configuration. When identified by morphology, membranes are often classified by their structures, being homogenous, heterogeneous, interpolymeric, symmetric or asymmetric [27]. In homogenous membranes, the ion exchange groups are attached directly to the base polymer structure so that the ionic charges are distributed over the membrane material [28]. Slight irregularities in local cross-linking or in densities of fixed charges may exist, however, they are considered to be chemically uniform [20]. Homogeneous membranes are the most desirable for many applications. Their properties will be most reproducible and they are often characterised by good electrochemical properties [28].

Heterogeneous membrane sheets are prepared by compressing a mixture of fine ion exchange granules and an inert elastic binder. The resins are embedded in the matrix irregularly [28]. The water-filled pores are formed by swelling the membrane in water. Ion exchange is a function of the exchanger particles; the binder only provides flexibility and mechanical strength to the membrane. The proportion of the binder used, depends on the ion exchange capacity (IEC), strength and selectivity of the membrane. Compared to homogenous membranes, the heterogeneous membranes have better mechanical strength, are easier to prepare but lack in good electrochemical properties [28]. Interpolymeric membranes are obtained by casting a film from a homogenous solution of two polymers, one of which represents the polyelectrolyte and the other being a water soluble filmogenic material [23]. Both components are strongly bound together so that immersion in water does not influence the polyelectrolyte. The interpolymeric membranes produced, possess properties of both the homogenous and heterogeneous membranes. 

Asymmetric membranes are commonly used in separation processes, often having high mass transport rates for certain components and good mechanical stability [27]. Asymmetric membranes are most commonly produced by the process of phase inversion (described further in Section 3). Phase inversion asymmetric membranes are composed of a thin, dense active layer with a porous support layer underneath. The combination of two or more membranes in series results in a composite membrane [29].

Development of membranes for redox flow cells has tended to mirror the work carried out in the area of fuel cell membranes. Membrane materials used for fuel cell applications can be separated into five categories [30]: (i) Perfluorinated ionomers; (ii) Partially fluorinated polymers; (iii) Non-fluorinated hydrocarbons; (iv) Non-fluorinated membranes with aromatic backbone; and (v) Acid-base blends.

**Scheme 1:**
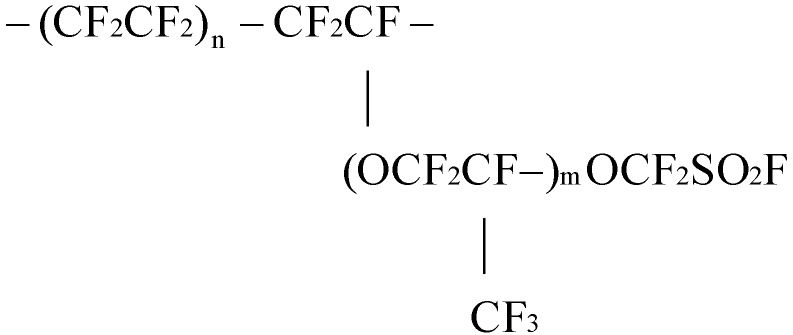
Structure of the precursor of perfluorinated ion exchange membranes [31].

Most conventional hydrocarbon ion exchange membranes degrade in the presence of oxidising agents. The perfluorinated membranes have been the most widely researched as they have the most desired properties of good chemical stability, high conductivity and mechanical strength. Perfluorinated ion exchange membranes are derived from copolymers of tetrafluoroethylene (TFE) and perfluorovinyl ether terminated by a sulfonyl fluoride group. The precursor of the perfluorinated membrane is shown in Scheme 1. The value of m may be 1 and n depends on the equivalent weight of the membrane. 

The sulfonyl fluoride groups can be hydrolysed to yield Nafion polymers in the form of sulphonic acid [31]. Here, the main chain is hydrophobic, whilst the acid or salt side chains are highly hydrophilic. Of particular importance is the perfluorosulfonic acid Nafion^®^ membrane produced by DuPont. In another class of perfluorinated membranes, the sulfonic acid groups are replaced by carboxylic acid. These include the Flemion^®^ (Asahi Glass Co. Ltd. Yokohama, Japan), NEOSEPTA-F^®^ (Tokoyama Soda Co. Ltd., Yamaguchi, Japan), and other Asahi Chemical Industry Company membranes. The Gore Select^®^ membranes (by W.L. Gore and Associates, Newark, DE, USA) [32] are micro-reinforced perfluorinated composites. They consist of an ultra thin microporous integral composite of a base material made of expanded PTFE whose pores are filled with the ion exchange material such as perfluorinated sulphonic acid resin or perfluorinated carboxylic acid resin [32]. A surfactant is usually applied to ensure impregnation. These membranes may also be reinforced with a woven or non-woven material bonded to one side of the base material.

Although perfluorinated membranes often possess properties desired in the battery industry, their application has until recently, been limited by a number of factors, the most significant being their cost (>$US800 for Nafion^®^ 117 [33]). Other disadvantages of these include safety concerns and temperature related limitations. Safety concerns arise from the evolution of toxic intermediates and corrosive gasses liberated at temperatures above 150 °C [30]. This is a concern both during the manufacturing process as well as vehicle accidents and could limit fuel recycling options. Degradation of membrane performance has also been observed at elevated temperatures, above 80 °C. 

Partially fluorinated membranes have a fluorocarbon base, with a hydrocarbon or aromatic side chain. The side chain is often grafted onto the backbone (to be discussed later) and is thus easily modified [30]. These membranes are relatively strong when compared to the perfluorinated membranes, but are less durable and likely to degrade faster. Hydrocarbon membranes are non-fluorinated, giving them inferior chemical and thermal stability. They are typically modified using polar functional groups. They have advantages in some applications over the perfluorinated as they are less expensive and therefore commercially available [30]. 

Hydrocarbon membranes can be modified by incorporating aromatic groups directly into their backbone or onto polymer groups in the backbone. This can enhance the stability of the polymer at elevated temperatures, and provide greater chemical stability in oxidising, reducing and acidic environments [30]. Acid-base blend membranes involve the incorporation of an acid component onto an alkaline polymer base [30]. This aids in promoting proton conduction. Improved thermal and chemical stability has been demonstrated in some short-term studies. A detailed analysis of some of these membranes can be found in Section 4.

## 4. Preparation of Membranes

The primary factors determining the properties of ion exchange membranes are the chemical nature of the polymer matrix and the functionality bound to this polymer material [34]. The secondary factors are determined by the method of manufacture. This controls the membrane morphology and has an important influence on the swelling behaviour and transport properties [34]. These primary and secondary factors will be reviewed in this section.

### 4.1. Method of Production of Ion Exchange Membranes

The fabrication of membranes is a multistage process, beginning with the production of the polymer followed by post-treatments to impart the selectivity and durability to the membrane [35]. Most polymer membranes are produced by conventional melt, dry or wet extrusion processes. Melt and dry extrusion is often the more feasible option due to the higher production rates than the wet process [35]. In melt extrusion, a polymer melt is extruded into a cooler atmosphere, which induces the phase transition, producing a membrane which is dense and isotropic [35]. A latent solvent (a compound miscible with the polymer at the extrusion temperature) may be added to produce a secondary phase separation. The removal of the solvent adds porosity. In dry extrusion, the polymer mixture is dissolved in a volatile solvent. Using an evaporation chamber, a porous isotropic or anisotropic membrane is created [35]. The wet extrusion method can be used to produce a greater variety of membrane morphologies. Here, the extruded mixture is coagulated by exposure to a nonsolvent in the form of a liquid or vapour (the basis of phase inversion). It is important to note that commercial polymers can display inconsistencies resulting from batch type production, even when obtained from the same manufacturer [35].

Other methods of membrane manufacture include the “paste method”, and membrane casting. The paste method [36,37,38] is used to produce the commercially available NEOSEPTA^®^ range of membranes (Tokoyama Soda Co. Ltd., Yamaguchi, Japan). The process begins with a paste consisting of a monomer with a functional group appropriate to introduce an ion exchange group, divinylbenzene (DVB), a radical polymerization initiator, and a finely powdered poly (vinyl chloride) (PVC). This paste is then coated onto a PVC cloth as reinforcing material, which is wound, together with separating film, onto a roll. The monomers are then copolymerized by heating to prepare the base membrane, onto which the ion exchange group is introduced. The ion exchange membranes produced by this method have been found to contain two continuous, finely and closely intertwined phases, the PVC and the ion exchange resin component [38]. 

Membrane casting is a method which produces ion exchange membranes by directly imparting the ion exchange functionality (such as sulphonic acid groups) to a base polymer material (such as polystyrene). According to this method [39]:

(i) Membranes are produced with relatively inexpensive materials.

(ii) The reaction is carried out in a homogenous liquid phase and thus the product is homogeneously sulfonated.

(iii) Application to large-scale processing is achievable. 

Ion exchange membranes are often symmetrical, homogenous membranes. Three general methods are used to prepare homogeneous ion exchange membranes [21]: 

(i) By the polymerization or polycondensation of monomers containing a moiety that can be (or can be made) cationic or anionic.

(ii) By the introduction of cationic or anionic moieties onto a polymer film.

(iii) By the introduction of cationic or anionic moieties into a polymer, followed by the dissolution of the polymer and casting it into a film.

Styrene and DVB are the most commonly used monomer starting materials for membrane polymerizations [19]. Cation exchange membranes are given their character by the attachment of the weakly acidic carboxylic acid groups, or the strongly acidic sulphonic acid groups [19]. Anion exchange membranes are produced by a two step process, chloromethylation followed by quaternary amination [19]. Strongly basic tertiary ammonium can then be added, whilst weakly basic groups include primary, secondary or tertiary amine groups. The preparation of anion exchange membranes is often more complicated and costly [19]. In addition, the chloromethyl ether used in the chloromethylation step is harmful to humans as it is classified as a carcinogen [40]. Several efforts have been made to avoid the use of chloromethyl ether, with one recent method using bromination instead of chloromethylation [41]. This method can also be applied to the preparation of cation exchange membranes. As mentioned earlier, properties such as low cost, ease of making and good electrochemical properties make heterogeneous ion exchange membranes more suitable to certain applications. Their mechanical strength however is comparatively low. By choosing a suitable binder or reinforcing material, the optimum combination of mechanical strength and electrochemical properties can be produced [21].

Ion exchange membranes have been produced by the interpenetrating polymer network (IPN) method, possessing excellent electrochemical and mechanical properties at a low cost [42,43]. The process involves the free radical polymerization of two monomers producing a chemical blend of two interpenetrating networks of linear and cross-linked polymers. This blend behaves like a homogeneous type of membrane having less probability of micro-voids compared to heterogeneous type membranes [21]. Semi-interpenetrating polymer network (sIPN) membranes have also been prepared by mixing two polymers followed by cross-linking with gaseous dibromoethane [44]. After the polymers are cross-linked, one of the polymers forms a network in which the chains of the second polymer are immobilised.

Asymmetric ion exchange membranes are often produced by phase inversion. In this process, a polymer solution inverts into a swollen, three-dimensional macromolecular network [29]. The production of these membranes involves five stages [45]:

(i) A homogenous polymer solution with required viscosity is produced.

(ii) The homogenous solution is then cast onto a suitable surface.

(iii) Part of the solvent is then evaporated off, resulting in the concentration of the polymer at the surface.

(iv) The polymer is precipitated in a precipitation bath, causing the exchange of the solvent for a precipitating agent, subsequently forming a gel.

(v) The polymer then undergoes tempering, where any imperfections on the thin layer are annealed (closed). 

During these five stages, changes to certain parameters can influence the membranes final structure and properties. These factors include the annealing temperature, the rate of solvent evaporation, the composition of the polymer solvent mixture and/or the composition of the precipitation bath [45].

The distinction of composite membranes to other asymmetric membranes rests with the mode of formation [46]. With phase inversion membranes, the polymer solution is cast in one step producing the microporous film with the thin dense layer one side. Composite membranes are formed in two steps. The microporous support is cast first and the barrier layer is then deposited on the support layer [46]. Composite membranes are a general improvement on phase inversion membranes as the support and active layers can be produced and optimised separately from a variety of materials for a particular function. Symmetric membranes can also be produced by the phase inversion process. In this case however, the precipitation agent is added to a gas phase, slowly, ensuring that the concentration remains constant over the whole film cross section. 

Graft polymerization is a process used for the modification of polymers [47]. Graft polymerization yields thin membranes with outstanding mechanical and good electrochemical properties [48]. A graft copolymer is a polymer which consists of one or more types of molecules as blocks connected as side chains to a main chain [49]. In the first step of the process, an active site is created in the polymer either by a free radical or an active group which initiates the polymerization leading to a graft copolymer. There are various ways to activate the polymer; by chemical, photochemical and high energy radiation. High energy radiation is the most versatile method as the grafting process can be more easily controlled. Although different types of high energy radiation exist (for example charged β particles and electrons), the most commonly used is the γ radiation source, Co-60 [49]. This technique makes it possible for two highly incompatible polymers to come together in the one material [21]. The degree of grafting corresponds to the percent increase in weight of the films after the grafting reaction by the following equation [49]:

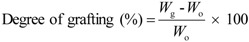
(1)
where *W*_o_ and *W*_g_ are the weight of the ungrafted and grafted membranes respectively. The specific grafting procedure is important in setting the membrane properties, as the polymer acquires some of the additional properties through the grafted component [47,49]. By varying the degree of grafting, it is possible to control ion exchange capacity, swelling and resistivity of membranes. Generally, as the degree of grafting increases, the number of ion exchange sites on a membrane also increases [49]. The increase in ion exchange sites on the membrane increases its swelling. 

Following grafting, ionic groups are introduced. Cross-linkers are also used in conjunction with the monomer to achieve certain properties in the grafted membrane [49]. Ion exchange membranes with a range of selectivities, resistivities and high mechanical stabilities can be made by choosing a suitable monomer and a cross-linking agent [23]. The technique overcomes the problem of membrane shaping, as the grafting can be done on a membrane film [21]. 

### 4.2. Fabrication of Microporous Separators

Unlike ion-exchange membranes, separators have no ion selectivity and function principally as physical barriers to prevent electrical short circuiting of the positive and negative electrodes while allowing the free movement of electrolyte to complete the circuit. Separators are a key component in many electrochemical systems, including batteries and fuel cells. Microporous membranes consist of a solid matrix with defined holes or pores with diameters ranging from less than 5 nm to more than 50 μm [27]. Microporous separators are often fabricated from inorganic and organic materials [50]. The simplest method of preparation involves the stretching of a homogenous polymer film of particular crystallinity. The stretching leads to a partial fracture of the film and relatively uniform pores [27]. This technique is commonly applied to polyolefin or polytetrafluoroethylene films [27]. Transport through these separators generally occurs by hydrodynamic flow, and hence the discrimination of specific species is not often possible. Selectivity through the micropores is obtained through the different transport rates of various ionic species [50]. Semipermeable microporous membranes have a slightly smaller pore diameter, and are able to restrict passage of certain ionic species. An interaction between the permeating species and the membrane may occur, and properties of selectivity of ion exchange membranes may exist. 

Ion exchange functionality may also be introduced to microporous separators by impregnating with soluble polyelectrolytes. The separator is immersed in the aqueous polyelectrolyte in which it swells and sorbs it [48]. The separator is then dried to trap the polyelectrolyte. Although this method of impregnation is relatively simple, during constant use, the capacity of such membranes has been known to fade [48]. This has been attributed to a lower chemical stability than ion exchange membranes [48]. 

## 5. Research Progress on Membranes for Redox Flow Batteries

In all the redox flow systems described in the previous sections, identifying and developing suitable membranes to provide the right balance of performance and cost have proven to be a major challenge. As identified earlier, the major problem with the iron/chromium redox flow cell was the cross contamination of the two half-cell electrolytes due to the passage of iron and chromium ions across the membrane. One method of overcoming this problem was the use of premixed Fe-Cr reactants in the two half cell solutions [51]. Other problems faced by Fe-Cr redox cell researchers included, the fouling of the anion selective membranes due to the formation of ferric chloride complexes [52]. Cationic membranes Nafion^®^ 117 and NEOSEPTA^®^ CR-2, as well as the non-selective, microporous separator Daramic (W.R. Grace) have also been tested. The Nafion^®^ 117 has produced the best result to date [53,54].

In the Regenesys S-Br flow cell, electrolytes are separated by a cation exchange membrane, which must allow sodium ions to be transported through in order to achieve electrical balance. It is important for the efficient running and lifetime of the cell that anions do not cross the membrane [55]. Sulphide diffusing from the negative to the positive side of the cell reacts directly with the bromine to produce sulphates that are not readily retrievable from the positive electrolyte and thus represent a net loss of sulphur from the system [56]. The challenge is to prevent anion diffusion without compromising the overall resistivity of the membrane. In vanadium redox batteries, a polysulfone based anion exchange membrane has been developed and used by both Kashima-Kita Electric Power Corporation and by Sumitomo Electric Industries in their initial 200kW x 4 hr rate demonstration system which has been operating since 1997 [57]. In a more recent study, an optimised 8 module 10 kW VRB stack incorporating a Nafion membrane, provided an overall energy efficiency of 82.35% at a charge/discharge capacity of 50 mA∙cm^−2^ [58]. In an attempt to highlight some of the significant developments in the synthesis and modification of membranes, the following sections provide an overview of the work on membranes that has been done in vanadium redox batteries, to date. 

### 5.1. Membrane Evaluation Methods

When evaluating membranes for use in the VRB, a number of tests are usually applied to establish the properties of each material in the vanadium redox cell. These tests include conventional measurements such as vanadium ion permeability, ion exchange capacity, ionic conductivity and area resistivity, chemical stability, water transport and cell performance during charge-discharge cycling in a redox test cell. Considerable work on the development of a range of test procedures for membranes for the VRB was carried out by Skyllas-Kazacos and co-workers [16,17,59,60,61,62,63,64] and these are now commonly applied by other research groups working in this area. 

#### 5.1.1. Vanadium Ion Permeability Measurements

The rate of diffusion of vanadium ions across the membrane will determine the coulombic efficiency of the cell and capacity loss with continuous charge discharge cycling. A standard test procedure was developed by Skyllas-Kazacos and co-workers [60,61,65,66] to eliminate any interference from water transport due to osmotic pressure effects across the membrane. Vanadium ion permeability measurements typically involve the measurement of the diffusion rate of V (IV) ions across the membrane from a 1 M VOSO_4_ and 2 M H_2_SO_4_ solution on one side, to a blank 1 M MgSO_4_ and 2 M H_2_SO_4_ solution on the opposite side using UV-Visible spectroscopy. MgSO_4_ is used in the blank solution in order to balance the osmotic pressure of the 2 solutions and eliminate the effect of water transport across the membrane[16,64]. Using Fick’s Law of Diffusion and Beer’s Law, the rate of V (IV) ion diffusion is determined by the rate of change in the absorbance according to the following derivation [64]:

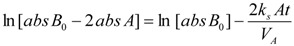
(2)
where *abs B_0_* is the initial absorbance of solution B (the 1M V (IV) solution), *abs A* is the absorbance of solution A (the 1M Mg^2+^ solution containing no V (IV) ions), *A* is the area of the membrane exposed, *t* is time and *V_A_* is the volume of solution A. The mass transfer coefficient (*k_s_*) of the V (IV) ions across the membranes was calculated from the slope of a plot of ln [abs B_0_ − 2 abs A] versus t. The linear plot produced has slope equal to *−*2k_s_A/V_A_ from which k_s_ can be determined. The diffusion coefficient, *D* is thus calculated using the following equation:

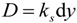
(3)
where *y* is the thickness of the membrane.

In a different study, Chen and co-workers proposed a method for determining the diffusion coefficients of VO_2_^+ ^and VO^2+^ ions across cation membranes at high electrolyte concentrations [67]. Using the Nernst–Planck equation, the potential difference across the membrane was calculated. For the three cation membranes that were compared, a linear dependence was observed between the logarithmic value of the selectivity coefficient and the molar fraction of vanadium ions in solution [67]. 

#### 5.1.2. Ion Exchange Capacity, Ionic Conductivity and Area Resistance Measurements

The ion exchange capacity of the membrane is determined by a titration method described by Sukkar and Skyllas-Kazacos [68]. The amount of H^+^ and OH^-^ is calculated from titration and the ion exchange capacity is calculated as the ratio of amount of H^+^ or OH^−^ to the weight of the dried membrane. The ionic conductivity of the membranes is determined by impedance spectroscopy and the measurements can be conducted in an area resistance test cell as designed in an experiment done by Vafiadis and Skyllas-Kazacos [17]. The test cell used, comprised of the membrane secured between two rubber gaskets, certain quantity of electrolyte in each of the half cells and two graphite discs covered with epoxy to act as electrodes. The same test cell can be used for determining the area resistance of the membrane. Resistance of the cell with the membrane (*r*_1_) and resistance of the cell without the membrane (*r*_2_) is measured and substituted into the following equation to calculate the area resistance of the membrane (*R*). A represents the area of the membrane. 


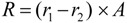
(4)

#### 5.1.3. Chemical Stability Measurements

Three different techniques have been employed to evaluate the long term chemical stability of membranes in the VRB. In the first method, samples of pre-weighed membranes are soaked in 2 M V (V) solutions and any weight-loss is periodically determined by simple weight measurements. A more accurate approach involves soaking preweighed sample of each membrane in 0.1 M V (V) solution. Oxidation of the membrane by the V(V) ions leads to the formation of the blue V(IV) species which can be used as an indicator to measure the stability of a particular membrane [62]. The concentration of V (IV) ions in the solution is determined by ultraviolet absorption spectrometry. The absorbance of each solution is periodically determined to monitor the rate of oxidation by V (V). In order to compare the stability of each membrane when employed in the vanadium redox battery, the experiment was repeated using a constant area (10.5 cm × 5.0 cm) of each sample exposed to 25 ml of the V (V) solution. To standardize the method, mixtures of 0.1 M V (IV) solution and 0.1 M V (V) solution are prepared with different ratios. The absorbance is determined for each mixture using a 0.1 M V (V) solution as reference for all measurements. The absorbance of each mixture was determined at a wavelength of 760 nm at which the maximum absorbance of V(IV) ions takes place [64]. In the third technique, cells incorporating the membrane under evaluation are subjected to continuous charge-discharge cycling for extended periods of time and the membrane resistivity and permeability are periodically determined to identify any changes due to membrane fouling or degradation during extended operation [62].

#### 5.1.4. Water Transport Measurements

**Figure 3:**
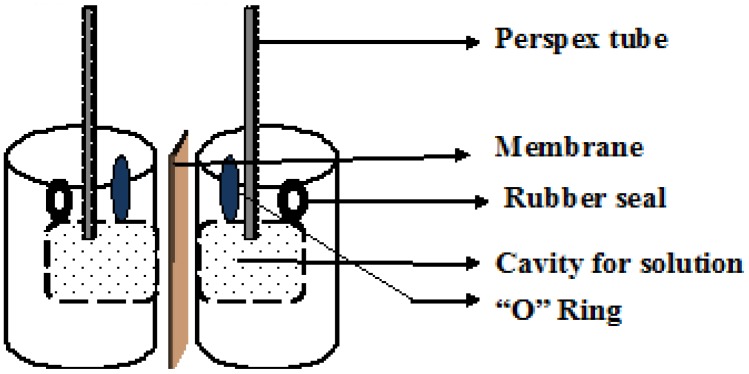
Schematic showing water transfer test cell (Adapted from [15])

In order to monitor the static water transfer across the membrane, a water transfer test cell was developed [60,61,65,66] as illustrated in Figure 3. The cell is typically constructed from clear perspex containing a 40ml cavity in each half-cell with the test membrane fixed between each half-cell cavity. A long perspex tube is attached to each half-cell cavity and solutions with compositions that correspond to specific states-of-charge of the two half-cell electrolytes are transferred into the cavities through the ports adjacent to the perspex tubes. The solutions are at the same initial level, about half way up the tubes and the level deviations attained by each solution are recorded periodically in order to plot height deviation as a function of time.

#### 5.1.5. Cell Performance Tests

The performance of the membrane in the flow cell is one of the most important considerations while selecting a membrane. To study the performance of different membranes, they are exposed to repeated charge/discharge cycles and their efficiencies are monitored. The performance of the cell is gauged in terms of coulombic, energy and voltage efficiencies. After several cycles of charging and discharging, the membranes are analyzed for stability and physical properties. An ideal membrane should exhibit good stability and capacity over extended periods of cycling [17,68]. 

### 5.2. Daramic (Microporous Separator)

Extensive work with the microporous separator Daramic has been carried out by several research groups. A low cost membrane fabricated from the modification of Daramic with Amberlite 400CG (an anion exchange resin) has been reported by Chieng *et al.* for use in the VRB [65,69]*.* The pores of the polyethylene microporous separator were partially blocked with a polyelectrolyte or an ion exchange resin while still imparting ion-selective capability to the membrane. Daramic was selected because of its chemical stability in vanadium electrolytes. Further cross-linking of the modified Daramic using divinyl benzene (DVB) showed a decrease in membrane diffusivity. When this membrane was employed in VRB cells, coulombic, voltage and energy efficiencies of 95%, 85% and 83%, were achieved, the efficiency values being much higher than untreated Daramic. A thin layer of poly (divinyl benzene) was shown to form after cross-linking, as confirmed from the area resistance, diffusivity, FE-SEM, TGA data and pore size distribution characterization [16]. In an attempt to impart cation-exchange capacity, the cross-linked Daramic was modified using poly (sodium 4-styrene-sulfonate) (PSSS) [66]. The composite membrane showed higher conductivity and vanadium ion diffusivity when compared to the anion exchange membranes employed in VRB system. This is because, in the case of cation exchange membranes, the current is carried exclusively by H^+^ ions, while the current is carried by both H^+^ and SO_4_^2−^ ions in anion exchange membranes. Thus, the low resistivity of the cation exchange membrane is due to an increased mobility of H^+^ ions. 

Another effort to impart cation-exchange capacity to the cross-linked Daramic by sulfonation using concentrated sulfuric acid showed promising results in terms of ion exchange capacity and net water transfer across the membrane [70]. It was found that the sulfonation reaction was able to incorporate cation exchange groups to the cross-linked Daramic. However, after sulfonation, only slight differences were found in terms of vanadium ion permeability, area resistance and cell performance. Daramic contains mineral oil which acts as a stabilizer, resisting oxidation. When Nafion was directly introduced into the pores of Daramic, it prolonged the open circuit voltage (OCV) decay and extended the time taken for self discharge [71]. 

### 5.3. Nafion (Perfluorinated Membrane)

Nafion is a sulfonated tetrafluoroethylene based fluoropolymer-copolymer which has excellent ionic properties. Nafion’s unique ionic properties result from the incorporation of perfluorovinyl ether groups terminated with sulfonate groups onto a tetrafluoroethylene (Teflon) backbone. The combination of the stable Teflon backbone with acidic sulfonic groups gives high cation conductivity as well as chemical stability. However, it is selectively and highly permeable to water [72]. 

Nafion has commonly been used as an ion exchange membrane in direct methanol fuel cell (DMFC) [73,74] and in VRB studies [75,76,77,78]. However with Nafion, there is a crossover of methanol when used in DMFC and vanadium ions in case of VRB, which results in a decrease in energy efficiency. While a few research groups have tried to find alternative membranes for VRB systems, that are available at a lower cost, most of the groups still use Nafion as a separator with several modifications to improve the stability. To reduce the methanol permeability in DMFC, surface modification using a layer-by-layer self-assembly polyelectrolyte [74] and in-situ sol-gel reaction (to incorporate inorganic oxide nanoparticles within the pores of Nafion [79,80] have been carried out. The following sub sections give an overview of the work that has been carried out by modifying Nafion membrane.

#### 5.3.1. Coating Nafion with Charged Substances (Polycations and Polyanions)

Interfacial polymerization was used to develop a cationic charged layer on the surface of Nafion 117 membrane in order to reduce the permeation of vanadium ions [81]. On comparing the modified membrane with unmodified Nafion, it can be seen that after modification, there is a dramatic reduction in the crossover of vanadium ions across the membrane and a slight increase in the resistance of the membrane. As a result, the coulombic efficiency of the VRB single cell (based on modified Nafion membrane (which is related to the concentration of the incubation solution of polyelectrolyte polyethylenimine (PEI)) increased significantly. The value was found to be 96.2–97.3%, which is higher than that obtained with the VRB single cell based on unmodified Nafion (around 93.8%). Due to a slightly higher area resistance caused as a result of modification, the voltage efficiency of modified Nafion is lower than that of pure Nafion. Furthermore, the water transfer across the modified membrane was also reduced. 

Another novel approach involved the fabrication of a barrier layer onto the surface of Nafion by alternate adsorption of a polycation, poly (diallyldimethylammonium chloride) (PDDA) and a polyanion, poly (sodium styrene sulfonate) (PSS) using a polyelectrolyte layer-by-layer self-assembly technique, which can suppress the crossover of vanadium ions [82]. The Nafion-[PDDA-PSS] n membrane (n = the number of multilayers) obtained, exhibited a much lower vanadium ion permeability when compared to the unmodified Nafion membrane. Moreover, VRB with Nafion-[PDDA-PSS] n as the membrane exhibits a higher coulombic efficiency (CE) and energy efficiency (EE) together with a slower self-discharge rate than that of the original Nafion system. Highest CE of 97.6% and EE of 83.9% can be achieved at charge–discharge current densities of 80 mA/cm^2^ and 20 mA/cm^2^, respectively.

Polyaniline/Nafion and polypyrrole/Nafion composite membranes were prepared by chemical polymerization and their properties were studied in the VRB [83,84]. The introduction of cationic polymers reduces the crossover of vanadium ions; however this modification also decreases the proton conductivity. It has been suggested that careful selection of the cationic polymer allows tuning the inherent trade-off between membrane permeability and resistance [84].

#### 5.3.2. Blocking Nafion with Inorganic Materials

Based on the pioneering work by Miyake *et al*. for DMFC applications [79,80], a Nafion/inorganic oxide nanoparticle hybrid membrane has been prepared using an in-situ sol-gel reaction. It exhibits a dramatically reduced permeability of vanadium ions and a lower self-discharge rate when compared with Nafion giving high coulombic, voltage, and energy efficiencies [85,86,87,88]. The inorganic oxide nanoparticles used were SiO_2_ [85,86], organically modified silicate prepared from the mixtures of tetraethoxysilane (TEOS) and diethoxydimethylsilane (DEDMS) [87], organic silica modified TiO_2_ prepared from the mixtures of DEDMS and tetrabutyl titanate (TBT) [88]. Nafion/TiO_2_ hybrid membranes were prepared by a hydrothermal method instead of in-situ sol-gel method [89]. It is reasoned that the polar clusters (pores) of the original Nafion become filled with the inorganic oxide nanoparticles during sol-gel reaction, which results in reduction in the cross-over of vanadium ions.

#### 5.3.3. Polymer Blending with Polyvinylidene Fluoride (PVDF)

Polymer blending is an effective method for polymer modification. Earlier, Nafion/PVDF blends were utilized to prepare cation exchange membranes for VRB systems [90]. The addition of the highly crystalline and hydrophobic PVDF seems to effectively control the swelling of Nafion. In VRB single cell tests, the Nafion/PVDF blend membranes exhibit higher columbic efficiency than recast Nafion at various current densities. The blend membrane with 20 wt% of PVDF shows an energy efficiency of 85% at 80 mA∙cm^2^, which is superior to that of recast Nafion. This membrane also exhibits a longer (twice) duration in OCV decay test and much lower permeation of V^4+^ when compared to recast Nafion. However, the mechanism behind the improvement in ion selectivity is still unclear [90]. 

### 5.4. Other Perfluorinated Membranes (PVDF as Backbone)

A solution-grafting method was employed to prepare a poly (vinylidene fluoride)-graft-poly (styrene sulfonic acid) (PVDF-g-PSSA) cation exchange membrane. The PVDF membrane was pre-treated with KOH and grafted with styrene via radical polymerization, followed by sulfonation. These membranes exhibited high conductivity and dramatically low vanadium ion permeability when compared to Nafion 117. The VRB with the low-cost PVDF-g-PSSA membrane exhibited a higher performance than that with Nafion 117 under the same operating conditions; its energy efficiency reached 75.8% at 30 mA/cm^2^. The performance of VRB with the PVDF-g-PSSA membrane was found to be stable even after 200 cycles at a current density of 60 mA/cm^2^ [78]. 

Another partially fluorinated cation exchange membrane was prepared by radiation grafting of styrene and maleic anhydride onto PVDF membrane followed by sulfonation. The grafted membrane showed a much lower permeability than Nafion 117. Moreover, the OCV was maintained above 1.3 V after 33 h, which was much longer than Nafion [91]. 

### 5.5. Non Fluorinated Membranes

Non fluorinated membranes have received broad attention in VRB due to their low cost, excellent mechanical and chemical stability and high ion selectivity [13]. Polyether ketone (PEK), Polyether sulfones (PES), polybenzimidazoles (PBI), polyphenyl quinoxalines (PPQ) are several families of thermoplastic polymers that are known for their excellent mechanical and chemical resistance properties at high temperatures. All of these polymers consist of 1,4-disubstituted phenyl groups separated by a number of linkages [92] which can be shown as –[X–(phenyl)–Y–(phenyl)–Z–(phenyl)]_n_, where X, Y, and Z represent –O–, –C (O)–, –C (CH_3_)_2_–, –SO_2_–, –S– groups. For PEK, X and Y could denote –O– whereas Z could be –C (O)–. For PES, X could be –SO_2_–, Y could denote –O–, *etc.*

Due to having benzene ring on their structures, -SO_3_H functional group can be introduced while fabricating an ion exchange membrane subsequently enhancing its ion exchange capacity and inducing good proton conductivity. However, the concentrated sulphuric acid used in sulfonation may cause decomposition of the polymers. Furthermore, due to high solubility of some sulfonated polymers in water, only a few of them can be converted into ionomers via sulfonation and fashioned into membranes that can be substituted for high cost commercially available perfluorocarbon sulfonates (e.g., Nafion). Sulfonated PES could only be prepared with a degree of sulfonation (DS) of 29.5% using concentrated sulphuric acid alone as the sulfonating agent. Attempts to sulfonate to a higher degree resulted in water soluble polymers that are unsuitable for membrane fabrication [92].

Polyether ether ketone (PEEK) is the most thoroughly studied, because it could easily be sulfonated into sulfonated PEEK (SPEEK) and could be produced with a wide range of sulphuric acid content [93,94]. The time and temperature of the reaction can be varied in order to achieve the desired level of sulfonation. Also, membranes based on sulfonated PEEK are promising materials for direct methanol fuel cell applications, exhibiting lower methanol permeability compared to Nafion membrane [95,96,97,98]. It has been reported that this was probably due to the difference in microstructures of Nafion and SPEEK. In Nafion membrane, the microstructures of polymers are composed of two parts: a highly hydrophobic fluorocarbon backbone and the highly hydrophilic sulfonic functional groups. This could give rise to hydrophobic/hydrophilic nano-separation, especially in the presence of water. The hydrophilic sulfonic groups cluster to form hydrophilic domains, which are well interconnected because of the high flexibility of the fluorocarbon of Nafion membrane. In SPEEK membrane, the backbone is less hydrophobic and the sulfonic acid group is less acidic which results in smaller hydrophobic/hydrophilic separation. Thus, the less pronounced hydrophobic/hydrophilic separation of SPEEK membrane produces narrow channels and a highly branched structure. 

A novel sandwich-type SPEEK/tungstophosphoric acid (TPA)/polypropylene (PP) composite membrane was tested in VRB by Jia and co-workers [99]. The sandwich membrane comprised of a layer of polypropylene (PP) (135 µm) between two layers of SPEEK/TPA membrane (total thickness: 100 µm). The Polypropylene membrane layer was immersed in a solution of SPEEK/TPA during the casting process. An extended stability of these membranes in VRB is based on the premise that even if the outer layer breaks, the other layers would still be able to perform the required function. The area resistance of the sandwich membrane was found to be higher than that of Nafion 212. The V(IV) permeability of SPEEK+TPA+PP is lower than Nafion because of the difference in the microstructures of SPEEK and Nafion, as mentioned in [100]. The V (IV) permeability of SPEEK+TPA is higher than the SPEEK+TPA+PP membrane because of a decrease in the pore size after SPEEK+TPA particles are embedded in the pores of the PP membrane which reduce the channel size for the diffusion of V (IV) ions. The permeability of SPEEK + TPA was also higher than Nafion 212 as a result of the hygroscopic nature of TPA that enables it to absorb water [99]. The SPEEK+ TPA+ PP membrane exhibited a higher average charge voltage and higher coulombic and energy efficiencies when compared to Nafion 212 and SPEEK+ TPA membranes. 

A Nafion/SPEEK composite membrane (N/S membrane) was synthesized and tested in VRB by Luo *et al.* [101]. The N/S membrane consists of a layer of SPEEK membrane between two thin layers of recast Nafion prepared by chemical cross-linking through the SO_3_H groups [101]. Nafion layer was designed to prevent oxidative degradation of non-fluorinated ion exchange membrane. Diamine was used to crosslink the sulfonic acid groups of Nafion and SPEEK. ATR-FTIR technique was used to confirm the formation of Nafion layer on the surface of SPEEK. The introduction of secondary amine groups neutralized the SO_3_H groups which resulted in lower Ion Exchange Capacity and a lower V (IV) ion permeability when compared to SPEEK. The N/S membrane was characterized by a higher area resistance, longer discharge time and a higher coulombic efficiency when compared to SPEEK and Nafion 117 [101]. 

### 5.6. Other Hydrocarbon Based Cation Exchange Membranes

Poly (arylene ether ketone), poly (arylene ether sulfone) and polyimide can be sulfonated and therefore can potentially be used as cation exchange membranes in VRB. A series of sulfonated poly (tetramethydiphenyl ether ether ketone) membranes with different degrees of sulfonation was prepared by direct aromatic nucleophilic substitution step polymerization. In this work that was carried out by Mai and co-workers [102], the performance of four different membranes namely, Nafion 115, SPEEK 40, SPEEK 50 and SPEEK 60 was studied in comparison to each other. It was observed that the ion exchange capacity increased with the degree of sulfonation. The area resistance of SPEEK was found to be higher than Nafion. This is probably because the conductivity is enhanced by the presence of SO_4_^2−^/HSO_4_^−^ ions in the pores of the membrane The V^4+^ and H^+ ^ion selectivity was highest in SPEEK 40 [102].

Sulfonated poly (fluorenyl ether ketone) membranes also known as SPFEK membranes, were synthesized directly via aromatic nucleophilic polycondensation of bisphenol fluorene with 60% sulfonated difluorobenzophenone and 40% difluorobenzophenone. Chen and co-workers studied and compared the properties and performance of Nafion 117 and SPFEK membranes [103]. Water uptake and swelling ratio was found to be higher for SPFEK when compared to Nafion. Because of a robust aromatic backbone, SPFEK has a higher tensile strength than Nafion 117. The permeability of V (IV) ions increased with temperature and time, and at a fixed temperature, the permeability was higher in SPFEK than Nafion. Also, the energy efficiency of SPFEK was found to be higher than Nafion [103]. In an attempt to improve proton selectivity while maintaining low vanadium ions permeability, a small amount of silica was introduced into SPFEK membrane by a simple sol-gel method [104]. The addition of silica enhanced the mechanical properties and thermal stability of the membrane. 

Sulfonated poly(sulfone) membrane was prepared by Kim *et al.* by sulfonating commercially available poly(sulfone) (Radel) with trimethylsilyl chlorosulfonate in tetrachloroethane, followed by casting the sulfonated polymer solution on a glass plate [105]. Sulfonated Radel showed lower permeability of vanadium ions than both Nafion 117 and pre-treated Nafion 117. The better ion selectivity of this membrane resulted in longer charge retention than Nafion 117. However, a decrease in Coulombic and energy efficiency after 40 cycles was observed, indicating problems in terms of stability[105]. Further study on stability of these membranes revealed that mechanical degradation during cycling might be due to local precipitation of solid particles inside the membrane. Chemical degradation was not significant when investigated by Raman spectroscopy [106].

Two other membranes, sulfonated poly (arylene ether sulfone) and sulfonated poly (arylene thioether) were synthesized via one-pot polymerization followed by sulfonation using chlorosulfonic acid as the sulfonating agent. Both membranes showed lower permeability of vanadium ions and a higher Coulombic efficiency than Nafion [107,108]. 

### 5.7. Anion Exchange Membranes

While it is difficult to prevent the cross-over of vanadium ions in the case of cation exchange membranes, it guarantees high proton conductivity and high energy efficiency. Meanwhile, using anion exchange membrane may significantly reduce the cross-over of vanadium ions through the membrane owing to Donnan exclusion effect, but it may not assist in the conduction of protons which decreases the voltage efficiency of the battery. Early studies of anion exchange membranes for use in the VRB were reported by Mohammadi and Skyllas-Kazacos [62]. Of the anion exchange membranes evaluated, Selemion AMV (Asahi Glass, Japan) showed relatively poor stability in the VRB during long-term charge-discharge cycling. In the case of the New Selemion Type 2 membrane however, the area resistance and diffusivity values remained almost constant after six months of testing, while weight loss was negligible after 2 months of soaking in V (V) solution. Further evaluation of modified Selemion AMV and New Selemion Type 2 anion exchange membranes was carried out by Mohammadi [59].

In more recent work by Hwang *et al.* [77], commercially available anion exchange membranes were modified by cross-linking through accelerated electron radiation. The cross-linked membranes exhibited better performance, indicating that cross-linking may improve the mechanical strength of the polymers. An anionic membrane, JAM–1-10 (JAM) was also tested and evaluated in the VRB by Tian and co-workers [109]. In comparison to other commercial ion exchange membranes, it showed the lowest permeability of vanadium ions and the best chemical stability in V (V) solution. When this anion exchange membrane was modified using poly (sodium 4-styrenesulfonate) by *in situ* polymerization, it resulted in an improved selectivity to cations [109]. 

To produce ETFE-g-PDMAEMA membrane, Dimethylaminoethyl methacrylate (DMAEMA) was grafted onto Ethylene tetraflouroethylene (ETFE) membrane by radiation grafting. This grafted membrane was then immersed in acid to improve the anion exchange capability in the form of –R_3_NH^+^. The optimization of dose used, or grafting yield was found to be important in producing the membrane with good area resistance [110]. As expected, the anion exchange membrane showed much lower permeability of vanadium ions when compared to Nafion 117. This is because, the positively charged quaternary ammonium salts repel the diffusion of positively charged vanadium ions. 

Poly (phthalazinone ether ketone/sulfone) also known as PPEK membranes, were prepared by chloromethylation, followed by quaternization to produce membranes with anion exchange properties. The membranes showed much lower vanadium ions permeability and a higher Coulombic efficiency than Nafion 117 [111]. The effect of amination agent on the properties of quaternized poly(phthalazinone ether sulfone) anion exchange membrane has been investigated by Xing *et al.* [112]. It was found that the addition of ethylenediamine could have improved the stability of the membrane.

### 5.8. Amphoteric Ion Exchange Membranes in VRB

By combining excellent properties from both cation and anion exchange membranes, novel amphoteric ion exchange membranes were prepared and studied in VRB by Qiu and co-workers [113]. Radiation grafting is one useful method to fabricate membranes with modified properties. A polyvinylidene fluoride (PVDF) membrane was grafted with styrene and dimethylaminoethyl methacrylate (DMAEMA) followed by sulfonation and protonation to produce a novel PVDF-g-PSSA-g-PDMAEMA with cation and anion exchange properties. The optimization of the dose used or grafting yield and DMAEMA content was found to be important in producing membrane with low vanadium ions permeability and good conductivity. Higher grafting yield led to higher water uptake, ion exchange capacity as well as conductivity. Meanwhile, higher content of DMAEMA resulted in lower conductivity and lower permeability. The amphoteric ion exchange membrane showed lower vanadium ions permeability than that of Nafion 117. This is due to the repelling effect of the positively charged quaternary ammonium salt and the positively charged vanadium ions that diffuse. The Open circuit Voltage (OCV) decay of the amphoteric ion exchange membrane was slower when compared to Nafion 117 [113]. 

In the same study, an ethylene-tetrafluoroethylene (ETFE) membrane was modified via a two-step radiation grafting process using styrene and Dimethylaminoethyl methacrylate (DMAEMA). ETFE-g-PSSA was initially synthesized by grafting the membrane with styrene; this was followed by sulfonation. This was done to prevent the decomposition of DMAEMA during sulfonation [113]. In another study by Qiu and co-workers [114], ETFE-g-PSSA was grafted using DMAEMA and then protonated in HCl, to produce a novel ETFE-g-PSSA-g-PDMAEMA amphoteric ion exchange membrane (AIEM). The AIEM exhibited lower permeability of vanadium ions and a high proton conductivity than Nafion 117. This could be due to Donnan exclusion effect because of which the charged cationic layer of the membrane repels the vanadium ions. The OCV decay was found to be much slower in the case of the amphoteric membrane when compared to Nafion 117 [114].

## 6. Water Transfer Studies

Preferential water transfer is an important issue to be considered while selecting a suitable membrane. Water transfer between two half cells can lead to precipitation of vanadium salts in the cell due to the concentration of one half cell electrolyte and dilution of the other. It can also cause flooding of the solution reservoir thus causing operational difficulties in commercial systems [63]. The water transfer behaviour of several commercial ion exchange membranes was initially studied by Chieng, Mohammadi and Skyllas-Kazacos [60,69] who found that there is a preferential volumetric transfer of the solution across the membrane during charge/discharge cycling in redox flow batteries, including VRB. It was observed that for a cell that employs an anion exchange membrane or a non-ionic separator, the net volumetric water transfer is towards the negative half-cell, whereas for a cation exchange membrane, the net volumetric water transfer is towards the positive half-cell [60,63]. The net amount of water transfer across a membrane is the summation of the water transferred by the hydration shells and the water transferred by osmosis.

In the case of cation exchange membranes, a significant amount of water transferred across membranes is caused by the hydration shells of V^2+^ and V^3+^ ions which carry large amount of water and can easily permeate through cation exchange membranes due to their relatively high charge numbers [60]. By changing the number and type of co-ions and counter ions in the membrane pores, different rates of transfer will be observed for each of the species in solution, so that the net water transfer direction would be expected to change, as would the net transfer of vanadium and sulphate/bisulphate ions across the membrane [63].

**Figure 4:**
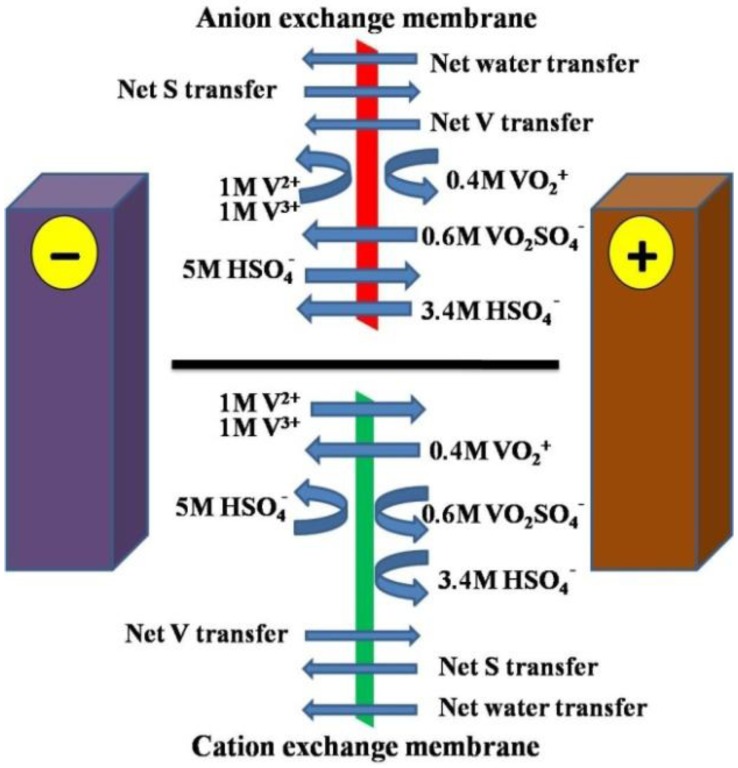
Expected movement of water and ions for Anion and Cation exchange membranes (Adapted from [15]).

Further water transfer study of cation exchange membranes has been investigated in terms of different state of charge (SOC) [63]. Interestingly, the direction of preferential water transfer is dependent on the SOC of the vanadium electrolytes. When the SOC is between 100% and 50%, the direction of water transfer is towards the positive half-cell, which is consistent with the study by Mohammadi *et al.* [60]. Further, when the electrolyte discharges from 50% to 0% SOC, the direction of water transfer reverses towards the negative half-cell. These studies are very important, because in long term VRB operation, huge water transfer can cause flooding of the electrolyte reservoir. Figure 4 depicts the movement of water and ions for a 2M Vanadium solution in 5M total sulphate at 50% SOC [15]. 

Chieng and Skyllas-Kazacos measured the transport number for water during charge-discharge cycling of the VRB using AMV, DMV, CMV, Nafion 117, and Flemion and treated Daramic and first observed a disparity in solution fluxes for anion and cation exchange membranes. A net accumulation of solution was observed in the negative half-cell electrolyte for anion exchange membranes, whilst cation exchange membranes showed the opposite trend. They proposed the use of alternate anion and cation exchange membranes in a cell stack to counter these processes and reduce if not eliminate the net solution transfer from one reservoir to the other [65]. 

In an effort to improve the water transport properties of commercial anion exchange membranes, they have either been sulfonated (the incorporation of some cation exchange capacity) or subjected to the addition of a cationic polyelectrolyte (poly (sodium-4-styrene sulfonate) [59]. The results show that sulfonation of an anion exchange membrane can incorporate some cation exchange capacity, which results in reduction of water transfer. The same mechanism is also applicable to the incorporation of cation polyelectrolyte into an anion exchange membrane. In a different study by Sukkar and Skyllas-Kazacos [15], different types of polyelectrolytes were used to modify cation exchange membranes by soaking them in a solution of the polyelectrolyte. This was expected to reduce the water transfer across the membrane. The results show that these modifications slow down the water transfer process and this may be due to the lower rate of diffusion across the modified membrane. However, it exhibits poor stability in the vanadium electrolyte which makes it unsuitable for continuous operation of a VRB. 

## 7. Vanadium Ion Transfer Studies

The transport of vanadium ions across the membrane is very important for evaluating the capacity loss in VRB. The nature of vanadium ion diffusion across ion exchange membranes provides an insight on how operating conditions can be optimized to reduce the crossover of vanadium ions and thus enhance the stability and performance of VRB. Chieng and Skyllas-Kazacos measured the diffusivity of each of the vanadium ions in the VRB across a range of membranes and found that these varied depending on the charge of each ion and the nature of the membrane. Table 1 summarises the results from this study.

**Table 1:** Diffusivity (cm∙min^−1^) of vanadium ions across different membranes [65].

Membrane	Diffusivity /cm min^−1^			
	V(II)	V(III)	V(IV)	V(V)
AMV	2.01 × 10^−6^	l.11 × 10^−6^	3.17 × 10^−5^	5.67 × 10^−5^
CMV	1.01 × 10^−5^	1.34 × 10^−5^	2.65 × 10^−5^	2.14 × 10^−4^
Nafion 117	4.63 × 10^−5^	4.24 × 10^−5^	1.94 × 10^−4^	1.41 × 10^−4^
Daramic (0.15mm)	6.21 × 10^−4^	7.09 × 10^−4^	1.46 × 10^−3^	>1 × 10^−2^
Composite membrane (0.23mm)	1.26 × 10^−4^	1.34 × 10^−4^	3.64 × 10^−4^	2.92 × 10^−3^

Generally, for all the different types of membranes investigated, the diffusivities of the V(IV) and V(V) species across the membranes were much higher than the V(II) and the V(III) species. The most pronounce difference was seen with the AMV anion exchange membrane. The higher diffusivities of V(IV) and V(V) species could be attributed to their ion pairing with the SO_4_^2−^ions, both negative and positive ion complexes having been reported. Complex formation of this kind thus reduces the efficiency of Donnan exclusion of the electrolyte, while for the V(II) and V(III) ions, no such complex formation has been reported to date. Importantly however, it is seen that different vanadium ions exhibit different diffusivities across all types of membranes, so that a net transfer of vanadium ions would be expected from one half-cell to the other during continuous charge-discharge cycling, this leading to an accumulation of vanadium ions in one half-cell and a dilution in the other half-cell. The consequence of this would be a slow loss of capacity that can however be restored by simply remixing the two half-cell solutions periodically [115].

The effect of vanadium ion diffusion on cell capacity was recently modelled by Skyllas-Kazacos and co-workers who simulated the changing concentration profiles of the different vanadium ions as a function of time over extended charge-discharge cycling in order to predict capacity loss due to the build up or decay of the different vanadium ions in each half-cell [116,117,118]. Results from the simulations using diffusion coefficient data for cation exchange membranes, were in agreement with empirical data for an operating VRB that displays a build up of vanadium ions in the positive half-cell during on-going charge-discharge cycling. The corresponding loss in capacity with cycle number is a function of the charge and discharge current used and the relative magnitude of the mass transfer coefficients of the four vanadium ions. 

Sun *et al.* studied the mass transfer of water and vanadium ions in charge-discharge cycles and a self discharge process by employing a kilowatt class stack based on Nafion 115 [119]. It was found that, in both the charge-discharge process and the charge-discharge cycles, the net transfer of vanadium is caused by the concentration difference of the vanadium ions between the positive and negative half cells. The transfer of vanadium ions with the bound water affects the transfer of water across the two half cells [119]. 

In another study by Vijayakumar and co-workers, a Nafion 117 membrane which is used in a vanadium redox flow battery, was analysed by X-ray photoelectron spectroscopy (XPS), nuclear magnetic resonance (NMR) spectroscopy and ultraviolet/visible spectroscopy [120]. Based on the results, the chemical identity and the environment of the diffused vanadium ions was studied extensively and analysed. From the UV/Vis Absorption spectra, it was seen that the V^4+ ^ions were adsorbed inside the nafion channel network and could not be removed by mechanical agitation unlike the other vanadium ion species. By analysing the surface deposition by XPS, it was revealed that the V^5+ ^ions were the predominant species on both surfaces of the membrane. Additionally, the NMR spectroscopic analysis showed Nafion to be chemically stable and there were no changes in the structure on exposure to vanadium electrolytes. Overall, it was concluded that the vanadium ions were not directly bound to the sulfonic acid groups in Nafion but they were bound to the water molecules in the hydration shell [120].

Another important consequence of vanadium ion diffusion in VRB applications was further highlighted in a simulation study by Ao *et al.* [121], who demonstrated the effect of the resultant self- discharge reactions on stack and electrolyte reservoir temperatures. Using vanadium ion diffusion coefficient values for Nafion 115 [119], they found that not only does the vanadium ion diffusion across the membrane lead to capacity loss as a result of different diffusion across the membrane and self discharge reactions, but that for the membrane in question, the magnitude of the heat generated by the self-discharge processes, could lead to excessive heating of the electrolyte, especially when the pumps were turned off during stand-by periods. Temperatures above 55 °C were predicted within the stack over the first half-day on standby, levelling off to the environmental temperature levels after 2–3 days. The study highlighted the importance of improved membranes to reduce self-discharge rate and thermal effects on the VRB. The researchers concluded that while Nafion 115 is extensively used in laboratory studies of the VRB, it is unsuitable for commercial systems where diffusion coefficients one tenth of the Nafion 115 values are required for adequate performance and temperature control during long-term operation [121].

## 8. Stability Studies of Membranes in Vanadium Solutions

The stability of a number of commercial and modified membranes was studied by Skyllas-Kazacos and co-workers in an early effort to identify and develop suitable membranes for the VRB [64]. The long term chemical stability of the sulfonated composite membrane (Daramic + Divinyl benzene) in V (V) solution was slightly poorer than cross-linked Daramic [62]. As a result of sulfonation, the poly divinyl benzene layer becomes thinner, which leads to lower chemical stability and a higher vanadium ions permeability. The long-term stability of commercial ion exchange membranes in VRB is limited by the oxidizing nature of the V(V) electrolyte, except Nafion 112 and New Selemion (type 2) [62]. However, further studies evaluating the chemical stability of several commercial ion exchange membranes have reported that Nafion 112 showed poor stability in the 0.1 M V(V) ions and a fair stability in 1 M V(V) ions when compared to several other membranes, different from the ones tested in [62]. The weight loss of the membranes is proportional to the conversion of V(V) ions to V(IV) ions, indicating that the chemical degradation is initiated by the oxidation of the membrane’s polyethylene backbone by V(V) ions in the positive half-cell electrolyte [68]. In the second generation vanadium redox batteries, as the Br^-^/Br_2 _redox couple is at a lower redox potential than the V(IV)/V(V) couple, it predominates. Also, the fact that Bromine is a highly corrosive element complicates the process of choosing a suitable membrane for the V/Br system [122]. 

## 9. Conclusions

Membranes for the VRB have been extensively studied. Although many membranes provide promising results, meeting all the requirements for the commercialisation of an economically viable system is still a challenge that requires further development in order to achieve the required cost structure for large-scale grid-connected applications. Membranes for the VRB must withstand the highly oxidative pentavalent vanadium ions in the case of the original VRB technology, or the oxidizing effect of bromine in the G2 V/Br system. While a large number of new membranes have been developed and successfully tested for use in the VRB, limited work has been carried out for the G2 V/Br system and for the more recently reported mixed sulphate/chloride VRB electrolyte [11]. Given that these systems promise significantly improved energy densities compared with the original VRB system, further testing of novel membranes in these electrolytes is warranted. Nafion continues to be one of the widely studied membranes in VRB systems; however its high cost, high levels of water transfer and high diffusivity values for the vanadium ions limit its use in commercial applications. New improved perfluorinated membranes possess good resistance to the oxidising V(V) and polyhalide ions, good mechanical properties and seem to be suitable for use in both VRB and G2 V/Br systems. The improved perfluorinated membranes have also demonstrated significantly reduced swelling compared with Nafion and much lower diffusivity values for the vanadium ions that significantly reduce self-discharge rates and potential thermal problems during stand-by periods when the electrolyte pumps are turned off [121]. Non fluorinated membranes which have been tested in the G2 systems so far, are not efficient because of the rapid self discharge reaction between the bromine and vanadium ions [14]. Hence, for the G2 and mixed acid flow battery systems, future work in membranes should focus on developing cost effective fluorinated membranes by experimenting with novel polymers and substances that can impart the required conductivity and chemical stability to the membrane. In the meantime however, novel membranes developed and shown to perform well in the G1 VRB electrolyte should be further evaluated in both the G2 V/Br and mixed acid electrolytes for possible short term application and scale-up towards commercialisation of these high energy density flow battery technologies. 
